# School-based smoking prevention programs with the promise of long-term effects

**DOI:** 10.1186/1617-9625-5-6

**Published:** 2009-03-26

**Authors:** Brian R Flay

**Affiliations:** 1 Department of Public Health, Oregon State University, Corvallis, Oregon, USA

## Abstract

I provide a systematic review of trials of school-based smoking prevention programs that had at least 15 sessions, preferably with some in high school, that reported significant short-term effects, and that included long-term follow-up. This is supplemented with a description of some other programs that produce short-term effects that portend large long-term effects. I conclude that school-based programs can have long-term effects of practical importance it they: include 15 or more sessions over multiple years, including some in high school; use the social influence model and interactive delivery methods; include components on norms, commitment not to use, intentions not to use, and training and practice in the use of refusal and other life skills; and use peer leaders in some role. School-based programs of this type can reduce smoking onset by 25–30%, and school plus community programs can reduce smoking onset by 35–40% by the end of high school. Some early childhood programs that do not have smoking prevention as their main aim, including home nursing, the Good Behavior Game, the Positive Action program and others, seem to change the developmental trajectories of children so that they are less likely to engage in multiple problem behaviors, including smoking, as adolescents. This review makes it clear that effective school-based smoking prevention programs exist and can be adopted, adapted and deployed with success – and should be.

## Background

Researchers and others have developed many school-based tobacco prevention programs over the past 30 years. Over a dozen reviews of approaches to tobacco control or substance abuse prevention published since the early 1990's have included school-based smoking prevention within their realm [1-17]. Some of these reviews were broad-based and non-systematic, and some were very systematic. Earlier reviews of this type always included school-based smoking prevention as a critical component of effective broad-based tobacco control. Many of the later reviews, especially after Lantz et al [7] tended not to include school-based prevention as an important component in broad-based tobacco control. Lantz et al [7] concluded that "The long term impact of school based educational interventions is of concern" (page 49). However, they then emphasize the need to combine school-based prevention with media programming, other tobacco control efforts, and other problem behavior prevention efforts. Dobbins et al [18] concluded that "there is reason for optimism regarding the effectiveness of prevention programs on smoking behavior and initiation, albeit in the short term." (page. 296).

During this same period there were many reviews [18-28] and meta-analyses [25,29-35] of school-based smoking prevention. These reviews and meta-analyses have repeatedly reinforced the fact that informational and affective programs do not work to change behavior. Furthermore, meta-analyses have further (established the fact that some psychosocial programs and strategies, particularly those that are interactive programs based on the social influences approach (educating youth about social norms and influences and providing skills for resisting such influences), can be effective.

However, findings in the field are sometimes confusing to practitioners and policy makers because some early or short psychosocial programs reported promising short-term effects that did not last [36-40]. In addition, some tested programs simply were not effective [41]. DARE is a prime example of a program that seems to be similar to many successful programs in many ways, yet it has been proven in multiple studies and two meta-analyses [42-44]. These mixed results have led some to question the overall value of school-based smoking prevention [45].

In an accompanying paper [46] I provided a review and critique of meta-analyses and systematic reviews of school-based smoking prevention that focus on long-term effects. Several of these reviews conclude that the effects of school-based smoking prevention programs are small and find no evidence that they have significant long-term effects. I found that these reviews all have methodological problems limiting their conclusions. These include severe limiting of the studies included because of performance bias, student attrition, non-reporting of ICCs, inappropriate classification of intervention approach, and inclusion of programs that had no short-term effects. The more-inclusive meta-analyses suggest that school-based smoking prevention programs can have significant and practical effects in both the short- and the long-term. Findings suggest that school-based smoking prevention programs can have significant long-term effects if they: 1) are interactive social influences or social skills programs; that 2) involve 15 or more sessions, including some up to at least ninth grade; that 3) produce substantial short-term effects. The effects do decay over time if the interventions are stopped or withdrawn, but this is true of any kind of intervention. In this paper, I provide a review of findings from prior meta-analyses and selected reviews, to be followed by a systematic review of the promise for long-term effectiveness of school-based smoking prevention programs.

## Methods for this Review of Long-Term Effects

My objective is to determine which kinds of school-based prevention can be effective over the long term and what their costs might be. This required a focus on studies of programs that both were successful in reducing smoking in the short term and also included follow-up data into high school (grades 10–12) or beyond. The general protocol of locating studies was through multiple sources; searches of PsycINFO, MedINFO, the Google Scholar search engine (using the terms "school," "prevent*," "tobacco," "smok*," and "program*"), and word of mouth. The duration of that search was from January, 1970 through July 2008. Any article or report in the English language that included data regarding the evaluation of school-based smoking prevention programs was included. Only studies with a control condition were included.

Since the purpose was to determine the size of the effects that could be obtained by the best programs that have been tested, the decision was made, based on the review of past reviews [46], to limit this review to programs that included 15 or more sessions (preferably including some in high school) and that had demonstrated effects at both short term and medium term. I did not limit this review to randomized trials, though most were. Only three school-based programs and four school+ (plus small media, mass media, and family or community components) programs fulfilled these criteria. These two sets of studies are labeled Category I studies of school-based and school+ programs, respectively. For Category I studies, only studies that included follow-up into high school were considered. Few studies included follow-up beyond high school, but for those that did, the reported effects are of interest. All seven Category I programs were included in the 25 studies with at least 2 years of follow-up reviewed by Skara and Sussman [11]. The other studies in their review id not meet one or more of the criteria for inclusion. For many, the last follow-up was earlier than grade 10 (and some of these are in my Category II). For some, there were no demonstrable short-term program effects [47].

Given the small number of Category I studies, evaluations of other programs with the promise of medium- and long-term effectiveness was also conducted. These Category II studies consist of school-based and school+ programs that had large effects that were of a large enough scope and sequence to suggest likely medium- and long-term effects. Ten studies of 8 programs met these criteria.

All of the studies reviewed in these two sections are from developed countries (the U.S., Finland and the Netherlands). Table 1 summarizes the categories and types of studies and programs in this review.

**Table 1:** Categories of studies and programs in this review

**Category I**	
High quality studies of	
Programs that were successful in reducing smoking in the short term, and	
Included significant effects into high school (grades 10–12) or beyond.	
***Two groups:***	
School-based only	
School plus community or mass media	
	
**Category II**	
High quality studies of	
Programs that were very successful in reducing smoking in the short term, and were	
Of a large enough scope and sequence to suggest likely medium- and long-term effects	
***Two groups;***	
School-based only	
School plus community or mass media	
	
**Category III**	
Any studies of	
Effects of smoking prevention in developing countries	

### Effect size

I use percent relative improvement (RI) as the indicator of effect size in this review for two reasons. First, it is readily available for all programs, whereas the detailed statistics needed to calculate an effect size are sometimes incompletely reported. Second, RI is readily understood and utilized in cost and benefit calculations. For randomized trials, pretest levels of smoking should be the same in both program and control groups, and RI would be the difference between posttest control (C) and program (P) groups divided by the control group level [i.e., (%C – %P)/%C]. However, pretest levels were not always the same, and these should be adjusted for; thus, in cases where pretest data were reported, RI is the posttest difference between groups minus the pretest difference between groups, divided by the control group posttest level, that is, (%ρC – %ρP)/%C_post_, expressed as a percentage.

One may ask how to compare the effect sizes reported in meta-analyses and RIs. An approach commonly used by meta-analysts [48] is to translate the effect size into a percentage reduction (RI) based on the area under the curve in the Z distribution. This approach translates an effect size of .20 into an 8% relative reduction in smoking. Programs with effects 2 to 14 times as great as this, that is, with RIs of 16–50% or corresponding effect sizes of between .4 and 2.8 were reviewed here.

Another complication in determining effect sizes is that different studies reported different levels of smoking as their outcome variable. For both short- and long-term effects, the most commonly used outcomes were ever (lifetime) use, use in the past month, or use in the past week. When studies reported more than one of these, I report them all in the tables. An additional level of complication is that some researchers report outcomes only for the subset of their sample who were nonsmokers at baseline/pretest. I noted reports on only those students who were nonsmokers at baseline in the text but not the tables. While relatively few studies reported more than one outcome measure, when they did so, the RIs were remarkably consistent across outcomes (with some exceptions). *On the assumption that investigators reporting only one outcome may have chosen to report the measure with the largest effect size, the estimates are likely to be biased upward*.

## Review of Category I Studies and Findings

To recap, Category I includes high-quality studies of programs that were both a) successful in reducing smoking in the short term and b) also reported significant follow-up effects into high school (grades 10–12) or beyond. For each of these programs, Additional file 1 shows the research design; the number of sessions, duration, and grade levels of the program, the grade of the last follow-up and the short and long-term program effects.

## Category I School-Based Programs

Reviews of evaluations of three school-based programs are included in this section

### The Tobacco and Alcohol Prevention Project (TAPP)

The Tobacco and Alcohol Prevention Project, developed by Bill Hansen and colleagues at UCLA in the early 1980s [49] was a 15-session social influences-oriented program. The core components of the social influences approach have been employed in many evaluated programs, including those reviewed here. Hansen [50] provides a good description of the theory and content of this approach. It has two main core elements: (1) resistance skills training to teach skills to resist the specific and general social pressures to smoke, and (2) normative education to correct student misperceptions of prevalence and acceptability of use. Programs using this approach also often involve active learning or the use of Socratic or dialectic teaching approaches, open discussion, the use of peers or older admired youth as instructors, and behavioral rehearsals to ensure that skills are learned well (subsequently called interactive approaches). TAPP included the above core elements plus inoculation against mass media messages, information about parental influences, information about the consequences of use, and making a public commitment not to smoke. Peer opinion leaders were used to assist teachers with program delivery.

TAPP was evaluated in two cohorts of seventh grade classes in a nonrandomized study in Los Angeles County. Only cohort 1, conducted in two moderately sized school districts, was followed into grade 10. Health education and social studies teachers received 2 days of training prior to delivering the program. As shown in Additional file 1, by the end of eighth grade the RI in past-month smoking was 26.2%. By the end of grade 10 there was a 19.1% RI in past-month smoking and 18.3% RI in ever smoking. In a secondary analysis of only those students present at all waves of the study, the RI in past-month smoking was 43%.

This was an early study of the social influences approach, and it demonstrated that the approach can be very effective. The use of peer leaders probably enhanced what program effects would have occurred with teacher-only delivery [51-53] The whole-sample result is preferred as the initial estimate of program effects because it provides a more realistic assessment of what would happen under real-world conditions; however, note that the larger effect obtained for students present throughout the study could be obtained if all schools were to implement the program.

### Life Skills Training (LST)

Life Skills Training is one of the most researched school-based substance use (including smoking) prevention programs. Developed by Gil Botvin [54], originally at the American Health Foundation and then at Cornell, LST consists of 30 classroom sessions with 15 delivered in grade 7, 10 in grade 8 and 5 in grade 9 (usually the first year of high school) (This is the number of lessons for the version tested in the studies reported here. Different versions of the program have different numbers of lessons per grade.) The program was designed to teach students a wide array of personal and social skills. These include content similar to other smoking prevention programs that focus on social influences [50,55], including learning and practicing refusal and other assertion skills, information about the short- and long-term consequences of smoking, correction of misperceptions of the prevalence of use by same-age peers, and information about the decreasing acceptability of smoking in society. Other generic program content addresses the development of communication skills and ways to develop personal relationships.

Multiple studies over 25 years have demonstrated the effectiveness of the program when delivered by different providers, in different kinds of schools, and for different kinds of students (see [56] and [57] for reviews). Only one study has included long-term follow-up through high school [58]. This was a follow-up of the largest single trial, conducted in 56 suburban and rural schools serving largely (91%) white students in three geographical regions of New York State [59]. Schools were assigned randomly to two experimental conditions (one day or video-taped teacher training) or a control condition. Level of implementation ranged from 27 to 97% by teacher reports, with about 75% of the students receiving 60% or more of the intervention. Six program schools and 18% of the students were excluded from the analysis of program effects because of poor implementation.

As shown in Additional file 1, at the end of grade 9 the RI was a relatively small 8.9% (1.63% versus 1.48%) for weekly smoking, partly reflecting the low prevalence of weekly smoking at this age. At the end of twelfth grade, the RIs were 19.7% (33% versus 26.5%) and 20.4% (27% versus 22%) for monthly and weekly smoking, respectively (Note that the RI of 21% [(33–27)/33] reported by Skara and Sussman was based on the method that used only posttest results. My RI is based on the method that includes pretest results). For the high-implementation group, the long-term RIs were both 28%. However, the RIs for the (almost) complete sample provide the most appropriate estimate of what effects could be obtained under real-world conditions – indeed, they may still be an overestimate of the effects that might be obtained when the program developer is not involved – although even larger effects might be obtained with full, high-quality, implementation.

Independent evaluations of LST have found similar short-term effects. In a nonrandomized trial in Spain, where the program was delivered by teachers to grade 9 students, a 21% RI in average monthly smoking at the end of grade 10 reduced to 11% by the end of grade 12 [60]. Independent evaluations of LST in Midwestern states found a short-term RI of 22% in a randomized trial in rural Iowa [61,62] and short-term RIs of 43% in current smoking Indianapolis [63]. Another small-scale (three schools per condition) randomized evaluation in Pennsylvania found small immediate effects for girls only, and these had decayed by the end of grade 7 and were no longer apparent by the end of grades 8–10 [64]. In a nonrandomized trial of a German adaptation of the life skills approach in 106 German-speaking elementary schools in Austria, Denmark, Luxembourg, and Germany, a 10% RI in ever smoking and less than 1% RI in past-month smoking were reported [65].

### Project SHOUT

John Elder and colleagues at San Diego State University [66,67] developed and evaluated Project SHOUT (Students Understanding Others Understand Tobacco), which used trained college undergraduates to teach 18 sessions to seventh and eighth graders that included information on the health consequences of smoking, celebrity endorsements on nonuse, the antecedents and social consequences of tobacco use, decision making, resistance skills advocacy (writing letters to tobacco companies, magazines, and film producers; participating in community action projects designed to mobilize them as anti-tobacco activists), a public commitment to not use tobacco, and positive approaches to encouraging others to avoid tobacco or quit. In ninth grade, five newsletters were mailed to students and two to their parents, and each student received four phone calls from trained undergraduate counselors that were individually tailored to their tobacco use status at the end of eighth grade or the prior phone call. During eleventh grade, approximately half of the students received two more newsletters that focused on tobacco company tactics to recruit new smokers', information on recent city, state, or national legislation regarding tobacco, cessation advice, and second-hand smoke; and one phone call that focused on eliminating smoking in restaurants and other public places, and the rights of customers and employees in those places affected by the potential ban.

The program was evaluated in 22 schools with ethnically diverse populations in the San Diego, California area, some suburban and some rural. Schools were assigned randomly to program and control conditions after matching on pretest levels of tobacco use. Effects observed at the end of 8^th ^grade (14.6% versus 10.8%, RI = 22%) were not statistically significant. However, as shown in Additional file 1, by the end of grade 9 the intervention produced a relative reduction in tobacco use in the past month of 30.3% (19.8% versus 13.2%). By the eleventh grade, the average RI was 44.1% (12.6% versus 7%). For the group that did not receive the grade 11 intervention, the RI decayed to only 9.5%.

The pattern of effects observed suggest that much of the long-term effect was due to personal attention via newsletters and phone calls in grades 9 and 11. Indeed, one has to wonder if the personal attention set up a response bias among respondents such that those who received personalized newsletters and phone calls were motivated to tell the researchers what they wanted to hear; however, lack of a differential response rate to the surveys by condition speaks against this, at least in part. Considerable research suggests that the power of similar-age peers and the power of college-age counselors for high school students should not be underestimated. Although the cost of the intervention as studied was kept down by the use of volunteer students, it is not clear how easily this model can be disseminated. The results also strongly suggest, however, that even a brief intervention during high school was enough to actually increase the effect observed at the end of grade 9.

### Category I School+ Programs

#### The North Karelia Project

Erkki Vartiainen and colleagues at the National Public Health Institute of Finland [68-71] tested a 10-session social influences program delivered by trained health education teachers and peer leaders in the province of North Karelia, Finland. A community-wide heart disease prevention program and mass media campaign modeled on the Stanford three-cities project [72] was going on throughout North Karelia at the same time. Two schools received the 10-session program from the project health educator and trained peer leaders and two schools received a 5-session version from regular teachers. Two schools from another province, where there was no prevention program, were used as controls. As shown in Additional file 1, at the end of grade 9 the RI (average of lifetime, monthly, and weekly) was 44.6% (for both program conditions), which decayed to 38.7% by grade 11. By 3 years beyond the end of high school, the RI had decayed to 22.9% in the health educator condition and 37.3% in the teacher condition; by 10 years beyond high school, the average RI was 20% with the two conditions not significantly different.

The results reported here can only be interpreted as the joint effects of the school-based smoking prevention program and the community-wide heart disease prevention campaign (which had a reduction of smoking as one of its targets). Thus, these results suggest effects that are larger than those of the school-based programs reviewed above. The larger effects obtained by regular teachers suggests that programs might be more effective when delivered by regular classroom teachers than when delivered by visitors to classrooms, possibly because of the ongoing relationships that teachers establish with students. However, at the long-term follow-up, teachers and visitors produced similar effects.

### Minnesota Class of 89

This project, conducted by Cheryl Perry and colleagues [73], then at the University of Minnesota, was another in which a school-based prevention curriculum was tested in the context of a community-wide heart disease prevention program. The community program consisted of community education, including mass media, and organization activities, including screening, cessation clinics, and workplace education, designed to reduce three cardiovascular risk factors – smoking, cholesterol levels, and blood pressure [74,75] The school-based smoking prevention program [76,77] was based on the Minnesota Smoking Prevention Program [78,79], one of the early social influences programs, and included material on diet and exercise as well as tobacco. Seven sessions on smoking prevention were delivered by peer leaders assisted by teachers in seventh grade. In eight and ninth grades an additional 10 sessions concerning tobacco use were delivered by teachers. The classroom components were supplemented by the development of health councils through which students participated in other cardiovascular risk reduction projects.

The smoking prevention program was evaluated with a design in which students in all of the schools in one community received both the community-wide cardiovascular intervention and the school-based smoking prevention program, and students in all the schools in another community did not. All students in one cohort were surveyed every year from grade 6 to grade 12. As in all school-based studies, attrition occurred continuously over the 6 years and, by grade 12 only 45% of the original participants were surveyed. There were no differences in smoking rates at sixth grade. By the end of seventh grade, after the core smoking prevention content had been delivered, weekly smoking prevalence was about 40% lower in the program condition, and this effect was maintained through twelfth grade (Additional file 1), 3 years after the end of direct smoking prevention instruction and a year after the end of general community education.

Like the North Karelia project, this study demonstrates that school-plus-community programming can have substantial effects that are maintained to a large extent through the end of high school.

### Midwestern Prevention Project (MPP)

The Midwestern Prevention Project (also known as Project STAR, Students Taught Awareness and Resistance), developed by Mary Ann Pentz and colleagues at the University of Southern California, tested a school-plus-community (and mass media) version of the social influences approach in eight communities in the Kansas City metropolitan area. The school-based component consisted of 10 sessions delivered by classroom teachers to sixth or seventh grade students (depending on the year of transition to middle school) and 5 sessions delivered the following year (when a parent involvement component was also implemented). Of these schools, 8 were assigned randomly to conditions, 24 other schools elected to deliver the program and 18 others elected to wait till after the project. Mass media programming was available to all communities every year. Other community-based programming started in the third year and likewise was available in all communities.

At the 2-year follow-up, the RI was 37.5% (Additional file 1; [80]). By grades 9–10, it was 18% (Additional file 1; [81]). These results are difficult to interpret because all students were exposed to the mass media and community components. The mass media programming, in particular, would be expected to reduce the difference between groups because the control group would no longer be a real control, and it might have reduced students' rate of onset relative to if they had not been exposed to the community program. This might explain the relatively fast "decay" that was observed.

### Vermont Mass Media Project

The Vermont project tested the effectiveness of a mass media social influences smoking prevention program when delivered in the context of a school-based program. Kim Worden and colleagues, of the University of Vermont [82,83], undertook a very careful development process to develop television and radio spots that would discourage cigarette smoking by adolescents. They randomly assigned two communities to the program (mass media plus school) condition and two matched communities to a school-only condition. There was no true control group. In the program communities, they purchased the time for airing the spots (734 TV spots in year 1 decreasing to 348 by year 4, and 248 radio spots in year 1 increasing to 450 by year 4) and provided schools with the school-based program (four sessions in each of grades 5–8 and three sessions in each of grades 9 and 10 – each student in the study cohort was exposed to 4 years of program during grades 5–8, 6–9, or 7–10) and teacher training to deliver them. Neither schools nor students were told about the media programming, and the mass media programming never mentioned the school program. Thus, as far as students were concerned, there was no linkage between the two programs.

As shown in Additional file 1, the RIs in weekly smoking among the school plus mass media program group compared to the school-only program group were 36.6% (14.8% versus 9.1%) at the end of the program (grades 9–11) and 28.8% 2 years later at grades 10–12 [82,84,85] Larger effects were observed for daily smoking – 44% RI at the end of the program and 36% a year later. It is difficult to estimate what the effects of the school-only program might have been, and, therefore, the relative contributions of the school and mass media programming. Nevertheless, this study demonstrates that well-designed media programming can produce large effects above those of a school-only program, about 80% of which are maintained for at least 2 years.

## Summary of Findings from Category I Programs

Results from three social influence and social competence programs with 15 or more sessions over 2–4 years, preferably with some content in high school, had significant long-term effects (i.e., at grades 10–12): an average of a 27.6% (range 18.7–44.1) relative reduction in smoking. The extraordinary effects of Project SHOUT may have been due to the added content on tobacco industry activities, the teaching and encouragement of advocacy skills, and the personal attention. These results need to be replicated. The long-term effects suggest that a minimal personal contact intervention of this kind in high school could increase the effects of any other program delivered in middle school. On the basis of these studies, I conclude that *social influences oriented programs of proven effectiveness, if well implemented, can produce a long-term RI of between 25 and 30% or an ES between .7 and .8*.

The school+ studies produced short-term RIs of about 40%, significantly higher than the school-only programs, consistent with my prior review [86]. These effects decayed over time an average of 22% to about 31% RI. Because the effects of school-only programs tended to increase rather than decay over time, the long-term effects of school plus community or mass media programs were only about 12% better than school-only programs. Note, however, that effects of programs that included a high school component (North Karelia and Minnesota Class of 89) were maintained at a higher level (almost 40%, or 31% better than school-only programs); reinforcing the conclusion above that high school programming reduces the decay of effects.

The use of multiple delivery modalities increases effectiveness over those obtained from school-only programs [86]. This is consistent with theories about the influences on behavior existing across multiple domains of life [87-91] It helps if students receive consistent messages across community contexts and over time.

Thus, we conclude that *ongoing school plus mass media or community programs can produce a long-term RI of between 35 and 40%% or an ES between 1 and 1.3*.

## Review of Category II Studies and Findings

This section provides a brief review of 10 studies of 8 programs that show exceptional promise or provide other important insights to help estimate the potential and likely relative reduction in smoking onset if prevention programs were widely implemented. These results are summarized in Additional file 2.

### The Adolescent Alcohol Prevention Trial (AAPT)

William Hansen and John Graham of the University of Southern California [92] tested two variants of early social influences programming (nine sessions delivered to grade 7 students) targeted to alcohol use. They contrasted information plus resistance skill training, information plus normative education, or both of these combined. Schools were assigned randomly to one of these three conditions or to a control. Although the program focused mostly on alcohol, it also produced effects on cigarette smoking. The normative education and combined programs produced the largest effects. As shown in Additional file 2, the RIs at the end of the program were 21.4% for lifetime smoking and 26.2% for monthly smoking. At eleventh grade follow-up, the RI in lifetime smoking was 13.9% [93].

Although this program focused mostly on alcohol, it also produced effects for cigarette smoking. These effects were not too different in magnitude from those reported earlier from TAPP (developed by the same principal investigator), although, as might be expected because the program was not focused on smoking, these effects were not maintained as well.

### Towards No Tobacco (TNT)

Steve Sussman and colleagues, also at the University of Southern California [94-96], developed the TNT program as a more intensive approach to tobacco prevention that incorporated the social influences approach and new approaches to altering normative beliefs and social skills training. In a large randomized trial, they found RIs in ever smoking of 34% at the end of the program (grade 8) and 30% at grade 9, and RIs in weekly smoking of 64% at the end of the program and 56% at the end of grade 9 (see Additional file 2). These effects are larger than those found in other programs, so one would expect that the long-term effects might also be larger.

### Know Your Body (KYB)

Investigators at the American Health Foundation developed the Know Your Body program in the early 1980s as a comprehensive health education program that included social influences and competence prevention components. It consisted of 384 lessons delivered during grades 4 through 9. In a randomized trial in two New York State communities, Walter and colleagues [97-102] found an 11.5% RI in thiocyanate (a biological marker of smoking) at grade 8. In one community, they found a 73.3% RI in lifetime smoking at the end of grade 9 (entered at half this value in Additional file 2, since it was not replicated in the second community).

In a second randomized trial with Washington DC African-American students, Bush and colleagues [103,104] obtained very similar results, 49.5% RI after one year and 80.8% RI after 3 years (at the end of grades 7–9). Again, these were based on saliva thiocyanate.

These are exceptionally large effects. Without long-term follow-up data we cannot be sure how well they would have been maintained, but these studies show that strong prevention effects can be obtained by comprehensive health education programs that also include proven approaches to prevention.

### The Good Behavior Game (GBG)

Shepard Kellam and colleagues at The Johns Hopkins University [105] applied the Good Behavior Game [106] to improving elementary student behavior in the expectation that it would prevent subsequent adolescent problem behavior. In a trial where grade 1 students were assigned randomly to classrooms, and classrooms or teachers were assigned randomly to the GBG, another intervention, or control conditions, students received three 10-minute sessions per day at the beginning of grade 1, increasing in frequency and duration during grades 1 and 2. Ialongo and colleagues [107] reported a 24% RI in problem behavior at the end of grade 2 and Fur-Holden and colleagues [108] reported a 26.3% RI in lifetime smoking at grade 8 (Additional file 2).

A second trial tested the effects of only one year of the GBG in grade 1 on smoking behavior 6 years later [109]. At the follow-up, students who had participated in the GBG were 21% less likely to have started smoking, a result remarkably similar to that obtained in the earlier study (Additional file 2).

These studies demonstrate that important changes in life course trajectories of behavior brought about early in life can lead to important changes in adolescent behavior, including smoking. Other school-based programs that improve elementary school children's behavior also have this kind of potential, for example, Fast Track [110], the Seattle Social Development Group program [37,111,112] and the Positive Action program (see below) Some non-school interventions that improve the behavioral trajectory of young children – for example, preschool home nursing visitation [113-115] – also have this potential.

### The Positive Action Program

The Positive Action (PA) program was developed by Carol Gerber Allred, a teacher and administrator, beginning in 1977 and expanded and revised multiple times since then based on process evaluation data and user feedback. It is a comprehensive character education program designed to improve character, pro-social and other positive behaviors and school performance as well as negative (problem or unhealthy) behaviors, including smoking. The program consists of developmentally-appropriate, 15-minute lessons, delivered by trained teachers on up to four days per week for grades K-12. In addition, the school principal and a PA coordinator/committee implement school-wide climate change activities to encourage and support positive behaviors by students. School counselors can provide additional lessons to high-risk students and parents can conduct weekly lessons with their children that parallel the school lessons. A community involvement component is also available. The program makes students (and their teachers and parents) aware of how they feel good about themselves when they do positive actions, how that good feeling can lead to further positive thoughts and actions, and that there is always a positive way to do everything. Students are taught what actions are positive in the physical (including engaging in health-promoting behaviors and avoiding harmful substances), intellectual (including motivation to learn, decision-making and problem-solving), emotional (including empathy, self-control, self-management and responsibility) and social (including getting on with others, refusal and other communication skills, and other prosocial behaviors) areas of their lives. Students learn the skills they need to perform all of these behaviors through lessons, discussion, role-play and practice. The program is very interactive [33,116]; interaction between teacher and student is encouraged through the use of structured class discussions and activities, and interaction between students is encouraged through structured or semi-structured small group activities, including games, role plays and practice of skills.

Three quasi-experimental evaluations used archival school-level data from Hawaii, Nevada and a large south-eastern school district in matched controlled designs [117,118] to estimate the impact of PA on school disciplinary reports and student test scores. Large effects were found for both kinds of data in all three studies.

Two randomized trials of PA have recently been completed, one in 10 matched pairs of Hawaii elementary schools and one in 7 matched pairs of Chicago elementary schools. After 3 years of PA, 5^th ^graders were 47% and 29% less likely to have started smoking than 5^th ^graders in control schools in Hawaii and Chicago, respectively [119,120] (Additional file 2).

The south-eastern quasi-experimental study [118] provided the only opportunity to date to examine the long-term effects of PA. The evaluation used school-level archival data from the middle and high schools that students from the studied elementary schools later attended. The middle and high schools had not implemented the program. Compared to middle schools with less than 65% PA graduates, schools with 65–75% PA graduates reported 16% fewer smoking incidents, and schools with more than 75% PA graduates reported 32% fewer smoking indigents (Additional file 2). Compared to high schools with less than 15% PA graduates, high schools with 15–27% PA graduates reported 10% fewer incidents of tobacco use, and schools with more than 27–50% PA graduates reported 20% fewer incidents.

These data establish that the effects of 4–6 years of PA in elementary school are maintained at high levels through middle and high school. It seems likely that effects would be even greater if students also were exposed to PA during middle and high school.

### School plus computer-tailored letters

Marlein Ausems and colleagues at Maastricht University in the Netherlands [121] tested the effects of a short (3 sessions) social-influence school-based program and 3 computer-tailored letters. The researchers developed a computer program to produce tailored letters to students using their baseline data on smoking-related attitudes, social normative beliefs and self-efficacy. The letters were mailed to students 3 weeks apart. Some schools provided the letters without the school curriculum.

Students in the letter-only and school plus letter conditions were 30% and 27% less likely, respectively, to initiate smoking by the 6-month posttest. By the 18-month posttest, students in the letter-only condition were 43% less likely to have initiated smoking (Additional file 2), but students in the school plus letter were only 14% less likely. The authors speculated that the simultaneous combinations of interventions might have caused an overload of information. However, it seems more likely that the school-based program was just not strong enough to produce the kinds of effects that longer school-based programs have produced. Perhaps a stronger school-based program followed by personalized letters would have produced larger effects like those reported by Project SHOUT [66].

### Project 16

Project 16, developed by Tony Biglan and colleagues at the Oregon Research Institute [122] was a randomized, multiple cross-sectional design to test the effects of a comprehensive community-based intervention designed to reduce smoking by seventh and ninth graders. Sixteen communities were assigned randomly to two conditions, a five-session social influences school-based program and the school program plus the community program. The community program included media advocacy, youth anti-tobacco activities, family communications about tobacco use, and reduction of youth access to tobacco. At the end of 2 years of intervention, the covariate adjusted prevalence of smoking among seventh and ninth graders in the community program communities had increased 0.9% (from 10.7% to 11.6%) while prevalence had increased 3.3% (from 8.1% to 11.4%) in the school-based only communities – leading to a RI of 21.1% (Additional file 2). One year later, the parallel rates were 5.9% (from 7.9% to 13.8%) and 2.1% (from 10.3 to 12.4%), respectively, or a RI of 27.5% (Additional file 2).

The RIs obtained by this intervention suggest that well-designed community-based interventions can have effects that seem likely to grow over time. The lack of a true control group makes estimating the true effect difficult; however, the results of this study suggest that significant medium- and long-term effects can be expected from well-designed and implemented school-plus-community programs. Perhaps a stronger school-based program accompanied by a strong community-based program could produce results that increase over time [86].

## Summary of Category II Programs

Although these programs are not strictly comparable, the average effect size of these 7 projects was 29% for short-term effects and 36% for medium-term effects (usually grade 8 or 9), but with large variation (12–50% for short term and 14–81% for medium term). The medium-term effects of these programs were larger than the long-term effects of Category I programs. Given that Category I programs actually had increased effects over time, these results suggest that long-term effects considerably higher than those derived from Category I programs may be possible with more comprehensive or newer school-based programs.

The results of the Good Behavior Game and Positive Action programs are particularly intriguing because they demonstrate the power of changing the trajectories of behavior early in life. A prevention program provided to these students in middle and high school might have much larger medium- and long-term effects on smoking and other health-related behaviors – but no evaluations of such combinations are available to date.

## Summary of Findings and Conclusion

### School-only Programs

This review suggests that interactive social influences smoking prevention programs that provide 15 or more lessons, start in upper elementary or middle school, and continue into high school can produce solid long-term effects. Other conditions that appear to improve the effectiveness of school-only programs relate to content (social influences and general social competence are of critical importance), how well they are delivered (related to how well teachers are motivated and trained), and the involvement of older peers. The findings are consistent with the 13 components of effective programs for elaborated by Nan Tobler as a result of her multiple meta-analyses [123].

Results from three social influence and social competence programs with 15 or more sessions over 2–4 years, preferably with some content in high school, had significant short-term effects of about 22% RI in monthly or weekly smoking that increased during high school in two of the studies to an estimated average of 28% RI. Some other programs (Category II) provided further evidence that (1) the social influence approach can affect tobacco use even when alcohol use was the main focus, (2) comprehensive health education programs that include strong social influence content can be effective, possibly even more effective than stand-alone social influence programs, and (3) programs early in life can alter developmental pathways for the better, including less tobacco use in adolescence.

Based on an average of the long-term effects of Category I studies, and supported by the estimated medium-term effects of Category II studies, I estimate that *the possible long-term (end of high school or age 18) effects of a national program of well-implemented, school-based smoking prevention programs of proven effectiveness could be as high as 25–30%*.

### School-plus-Community and/or Mass Media Programs

The four Category I school-plus-community studies produced short-term RIs of about 40%, decaying to long-term effects of about 31%. As noted earlier, program effects were maintained at a higher level (almost 40%, or 31% better than school-only programs) for those programs that included a high school component (North Karelia and Minnesota Class of 89). Thus, *the possible long-term effects of a national program of well-implemented school- plus-community and/or mass media smoking prevention programs of proven effectiveness could be as high as 35–40%*.

### Expected Effects into Young Adulthood

*Program effects are likely to decay beyond high school*. Unfortunately, few studies are available to guide us in how large or small this decay might be. National U.S. data may allow for an estimate, though it is unclear how applicable it will be in other countries. The U.S. National Household survey on Drug Abuse data suggest that about 3.012% (average for 1989–1999, range = 2.63–3.46) of 18 year-olds who are not smoking daily become daily smokers by the time they are 25 [124]. The Monitoring the Future 2003 data provide a national estimate of the percentage of grade 12 students that smoke daily at 15.8%, meaning that 84.2% of twelfth graders were not smoking daily. For school-only programs, this would represent a 24.9% RI in daily smoking by age 25 (see Table 2 for calculations), with a decay in RI of (30 - 24.9)/30 = 16.9%. For school plus ongoing community or media programs, the comparable figures would be a 33.2% RI in daily smoking by age 25 (see Table 2), with a decay in RI of (30–33.2)/30 = 16.9% (coincidently the same as for school-only programs). In reality, I expect that the decay of school-only programs might be greater than this estimate, perhaps closer to 20%, and the decay of school plus ongoing community or mass media programs might be less, say 15% because the messages remain in the larger environment to influence or reinforce behavior. Of course, it is very difficult to draw any conclusions about long-term decay in developing countries.

**Table 2:** Calculation of decay in prevention effects by age 25

**Average school-only RI =**	30.00%
**Average school + community/media RI =**	40.00%

	

Without the prevention	

Average proportion not smoking in high school who will start by age 25 (SAMHSA Household Survey 1989–99)	3.12%

Average high-school daily smoking without intervention (Monitoring the Future, 2003)	15.80%

Proportion of new smokers by age 25 =	2.63%

Therefore, total proportion smoking by age 25 =	18.43%

	

With school-based prevention	

Proportion smoking after school-based prevention =	11.06%

Therefore, proportion not smoking =	88.94%

Therefore, proportion new smokers by 25 =	2.77%

Therefore, total proportion smoking by age 25 =	13.83%

**Therefore, new RI =**	**24.9%**

Decay in RI =	16.9%

	

With school + community/media prevention	

Proportion smoking after school-based prevention =	9.48%

Therefore, proportion not smoking =	90.52%

Therefore, proportion new smokers by 25 =	2.82%

Therefore, total proportion smoking by age 25 =	12.30%

**Therefore, new RI =**	**33.2%**

Decay in RI =	16.9%

## Expected Effects and Costs

### Expected Effects under Real-World Conditions

There are at least two other factors that could reduce the effects of even the best programs in real-world implementations (1) rate of adoption by schools and communities, and (2) level and quality of implementation or delivery.

Active adoption of a program does not necessarily translate into high implementation of it. For example, among Oregon schools who obtained funding to implement smoking prevention in 2001 (from Master Settlement funds), 37% implemented at a very low level and produced less than 1% RI in student smoking when compared with control schools (who did not obtain smoking prevention funding); another 39% implemented at a medium level, producing an 11.5% RI; and 24% implemented at a high level, producing a 48% RI in student smoking [125]. These figures would lead to about a 25% RI overall if one combined the medium and high implementation results, or only 16% RI if one averaged over all schools who obtained funding. *Clearly, less-than-complete implementation could dramatically reduce the expected effect size of any national effort*.

Experience in the USA suggests that getting effective prevention programs adopted (and used) by schools is not easy [4,126,127]. Estimates of effects often come from efficacy trials in schools or communities willing to adopt the program have been entered into the study where implementers are trained and monitored by the researchers. It would be helpful to have an estimate of the proportion of schools that would be willing to implement an effective tobacco prevention program; however, we know of few such estimates. As an example, the Conduct Problems Prevention Research Group [110] reported that seven of eight school districts that were offered the Fast Track program accepted, and 52 of the 54 schools asked agreed to participate.

In actuality, not even all schools entered into studies always carry through with their willingness to implement the program. For example, we noted above how 6 program schools were dropped from the LST analysis because of lack of implementation [59]. In another example, Battistich, et al. [128] reported that only 5 of 12 schools recruited into the program arm of a nonrandomized project based on faculty interest and perceived likelihood of being able to implement the program actually implemented the program moderately well to very well during the 3-year study.

In these days of high demands on schools, they are unlikely to address prevention unless they have to, or unless it can be shown to also improve achievement, and they are unlikely to adopt a program unless they have the funding for it. Adoption probably would not be 100% even with a clear mandate and earmarked funding, although it might increase over time, following the standard S-shaped adoption curve as successes are publicized [129]. *A clear mandate to include tobacco prevention in the curriculum together with earmarked funding and monitoring of adoption could help obtain rates of adoption of evidence-based school-based programs of up to 75% or more*.

Getting comprehensive programs implemented fully and with integrity, even when they are adopted with full information and commitments, also is no small task, and the level and quality of implementation are clearly related to program effectiveness [130]. Factors believed to influence program implementation have been identified, and they are related not only to the program itself (e.g., program complexity, provision of technical assistance, user- friendly materials) but also to the environment in which the program is implemented (i.e., district, school, teacher, and participant characteristics) [131].

For some programs with high levels of monitoring, levels of implementation might be high. For example, the Conduct Problems Prevention Research Group [110] reported that participating teachers taught an average of 85% of the lessons in the first year of the program, 91% of parents participated in the program, and 79% of them attended at least 50% of the parent sessions.

Without ongoing monitoring, implementation might be much more uneven. Uneven implementation of a national program could reduce the effect size substantially – but by how much? The effect sizes reported for LST already took incomplete implementation into account. The authors reported that about 76% of the students received 60% or more of the program from trained teachers in schools who had signed onto the study [58]. The 20% long-term RI reported was for the whole sample (for the high-fidelity sample, the long-term RI was 28%). Independent evaluations of the LST program have reported a wide range of effects; however, none of these studies provided data on levels or integrity of implementation.

The tobacco industry has sponsored adoption, implementation, and evaluation of LST [132,133] (Unfortunately, the design of this evaluation (unmatched control group, for which date are not reported) does not allow for any interpretation regarding program effectiveness).(During the first 2 years, teachers who provided implementation data (73%) taught 80% of the units, met 75% of the objectives, and covered at least 69% of the activities. If one assumes that the 27% who did not provide implementation reports did not teach LST, then the average implementation level would be between 50 and 60%. Some teachers noted that the only reason they implemented LST at all, especially in year 2, was because it was being monitored or evaluated. Thus, one could conclude that *under conditions of ongoing monitoring or evaluation a high level of implementation (60% or more) could be achieved*.

There may be less compromise in the delivery of a community or mass media campaign than of school programs because they are of larger scale. A 75% implementation level might be a reasonable expectation for well designed and fully funded (including purchase of time on television and radio) campaigns.

## Cost-Effectiveness

The final objective of this review is to estimate the costs and cost-effectiveness of school-based smoking prevention. It is difficult to estimate the costs and the value of benefits of successful prevention programs and, therefore the benefit/cost ratio [134,135]. There are at least two reasons for this: 1) the costs and benefits are so variable and 2) the long-term effectiveness of prevention programs has been so variable [136]. Nevertheless, several scholars have provided estimates.

One analysis estimated the cost of an effective 30-session prevention program in the U.S. at $US150 per student for program materials, training, teacher time and other costs [134]. However, the estimated savings due to the benefits of preventing significant numbers of students from starting to smoke, and delaying the start date (and therefore the lifetime consumption) for others, are significant [137]. For example, the estimated social benefits of smoking prevention alone are about $US300 per student, a benefit/cost ratio of 2.0, and the estimated total benefits are about $US840, a benefit/cost ratio of 5.6.

The cost of an effective smoking prevention program in Canada has been estimated at $CAN67 (1996 dollars) [138]. Assuming a modest 4% long-term effectiveness, benefits of smoking prevention were estimated to be lifetime savings on health care of $CAN3,400 per person and on productivity of almost $CAN14,000 [138], a benefit/cost ratio of 15.4.

Scholars disagree on whether lives saved actually save money or add costs for society. According to Hodgson [139], smokers incur about $9,379 more in lifetime health costs than nonsmokers. Using this information, Wang and colleagues [140] estimated the cost-effectiveness of LST to be about $US13,316 per life saved and $US8,482 per QALY (with the program costing only $US13.29 per student).

Another group estimated the costs of Project TNT at $US48 per student and that TNT would cost about $US20,000 per quality-adjusted life-year (QALY) gained [141]. While not cost saving, they concluded that smoking prevention offers gains in both survival and health-related quality of life that make it worth the cost. This latter statement is based on citizens' "willingness to pay" for gains on the order of several hundred thousand dollars per QALY saved. In contrast, another analysis found that the median cost of 587 medical and public health interventions is $US42,000 per year of life saved [142], so can be conclude that *smoking prevention is more efficient than most health/medical interventions*.

The social benefits of even broader behavior improvement programs could be considerably greater. Steve Aos and his colleagues at the Washington State Institute for Public Policy conducted an analysis of the cost-effectiveness of about 70 different prevention programs [143]. They estimated that LST costs $US29 per student and leads to benefits of $US746 (due to both smoking and drug prevention), a benefit of over $US25.61 per $ spent or a benefit/cost (b/c) ratio of 25.61. They estimated that TNT costs only $US5 per student and produces a benefit of $US279, a b/c ratio of 55.84. Other programs included in both that review and the current one include the Good Behavior Game (b/c = 25.92), the Midwestern Prevention Project (5.29), the Minnesota smoking prevention program (102.29) and a category of "other social influence/skills building substance prevention programs" (70.34).

*Clearly, from a societal perspective, the costs of effective prevention are well worth it both to the individual students and to society as a whole, at least in developed countries*. In developed countries, cost-benefit estimates ranged from 2 to over 100. It is difficult to determine the likely cost-benefit in developing countries, but the high costs of program delivery in developed countries combined with the high social and health costs of not preventing tobacco use in developing countries, suggest that prevention would also be cost-effective in many developing countries.

## Limitations

There are at least 10 limitations to the studies that met the criteria for this review.

1. Many prevention studies – including some of those reviewed here – did not use randomization, but instead used matched controls or other designs. Some so-called quasi-experimental designs [144] may be acceptable under certain conditions [145]. *The most appropriate design for evaluating school-based prevention programs is the school-based randomized trial*, where schools are assigned to conditions and data are analyzed taking into account the nesting of students in schools [146-148].

2. Although more than one program has reported significant long-term effects, none of the individual programs has more than two evaluations of long-term effects. Thus, although we can conclude that comprehensive, interactive programs, using the social influences approach, with 15 or more sessions, including in high school, can have long-term effects, we do not yet know whether the long-term effects of any one of the programs meeting these criteria can be replicated. *Replication studies, especially in different cultural groups and countries, are urgently needed*.

3. There is a reliance on self-report measures of tobacco use. For many years, the validity of self-reports of sensitive behaviors was questioned. After a series of studies of the use of biochemical validation or the collection of biochemical samples for use in a "bogus pipeline" procedure [149-151].(, methods for surveying adolescents that ensure confidentiality were developed that seem to ensure the validity of self-reports of sensitive behaviors [152-155]. Although multiple studies suggest that students do report their substance use honestly when asked under conditions of confidentiality, these studies were limited to middle school students in developed countries, so *it would be wise to have some studies with different ethnic groups and in developing countries use biochemical verification of self reports of smoking *with high school students and young adults.

4. The available long-term evaluations do not allow determination of the relative effectiveness of these programs for different populations. However, indications from meta-analyses that these types of programs have larger effects in schools with a predominantly special or high-risk (minority, high absenteeism or dropout, poor academic records) populations are promising. *We need more replication studies with different populations, within both developed and developing countries*.

5. The last time of data collection in most of these studies was while youth were still in high schools. *We need many more truly long-term studies of the ongoing effects of smoking prevention programs, preferably up to age 25*.

6. There is great variability in the way researchers and evaluators assess outcomes. Researchers have used ever smoking, smoking in the past month or week, and other indicators of youth smoking. Some researchers included only baseline never smokers in their analyses. Fortunately, there was reasonable consistency in estimates of prevention effectiveness across measures in most of the reviewed studies. Nevertheless, it would help future reviewers if researchers could settle on consistent measures or report results for multiple measures. In addition, however, future research needs to include assessment of multiple short-term effects (or mediating variables) in addition to tobacco use. For example, programs are designed to improve knowledge of the influences on behavior (including tobacco industry promotions); knowledge of the physical, economic, environmental and social consequences of tobacco use; perceptions of risk; normative estimates or beliefs; decision-making, peer pressure resistance, and coping skills; and possibly student's activism against smoking in their environment. All of these *mediating variables need to be measured in future research, and their mediating effects on tobacco use behavior demonstrated*.

7. There was large variation across studies in program content, which affects the validity of some prior reviews of this literature. Conducting meta-analyses of these studies seems like comparing apples with oranges, or even with yams (instead of comparing multiple crops of Gala apples or even different breeds of apples). The variation makes it difficult to compare programs. In other disciplines, one would not conduct a meta-analysis or review of such different kinds of programs and draw a conclusion for all programs as a group. One would not, for example, conduct a meta-analysis of all treatments for breast cancer and conclude that breast cancer treatment does not work. Rather, one would attempt to determine which kinds of treatments work the best (and for whom and under what conditions), and then adopt the best treatment as the standard of practice. Unfortunately, some meta-analysts of various smoking prevention programs have treated them as a homogeneous group and concluded that they are not very effective, and that they do not have medium- or long-term effects. It would be more appropriate to *try to find which kinds of programs produce significant effects, or the largest effects (as well as for which kinds of people and under what conditions)*, as Tobler et al. [33], Hwang et al. [31], and this report have attempted.

8. We still lack consistency and continuity across developmental stages (preschool through college), and this clearly is an area where continued research is desirable. At the preschool and elementary school levels, implementation of more general and promising approaches such as the Good Behavior Game or the Positive Action program should be used to prepare students to adopt tobacco-free lifestyles. Increasing evidence suggests that behavior improvement or positive youth development programs can have pervasive effects on behavior, including reducing tobacco use, and also can improve school performance. However, the lack of replicated findings regarding specific effects on tobacco use to date suggests that they should be accompanied by rigorous evaluations. Such *evaluations of general behavior-improvement and youth development programs will contribute to the knowledge base of smoking prevention as well as youth development*.

9. Program developers were involved in all of the evaluations reported. It is quite probable that the effect sizes reported when program developers are involved were larger that those that will be obtained under other conditions. *The field is urgently in need of independent replications of the findings summarized in this paper *[145].

10. There is a relative dearth of rigorous research on school-based smoking prevention in developing countries. However, several countries are making progress with the help of researchers from more developed countries [27,156-168]. *Much more research on school-based smoking prevention in developing countries is urgently needed*.

Despite, or maybe because of, the above limitations, there are multiple reasons to suspect that estimates of effect sizes derived from the small number of studies reviewed here (RI of 25–30% for school-only programs or 35–40% for those that add on-going community activities or mass media campaigns) might be conservative (under) estimates of effects that might be observed under ideal conditions. First, some of the effect sizes were derived from studies that already included less than optimal implementation. Second, if a program was implemented nationwide, for multiple years, there might be increasing effects for a while as new generations of students passed through the program. For example, as fewer young adults become smokers, there will be less social support for smoking and fewer adolescents will be tempted to try smoking. Third, the possibility of larger effect sizes were suggested by the larger short-term effects of the Towards No Tobacco and Know Your Body projects, the promising effects of general behavior improvement programs such as the Good Behavior Game and the Positive Action program, and the extraordinarily large effects of Project SHOUT with minimal high school boosters.

The current research demonstrates a phenomenon of decreasing effect sizes over time (see [33] Figure 9). For example, in the Tobler et al meta-analyses, the average effect size decreased from .18 in the 1997 report [34] to .15 in the 2000 report [33]. One reason for this might be that as more and more tobacco control activities occurred, true control groups became less and less likely.

## Conclusion

This review makes it clear that effective school-based smoking prevention programs exist and can be adopted, adapted and deployed with success. Communities and school districts should invest only in these research-proven programs and avoid spending money on programs where there is little or no prior evidence of program effectiveness. When implementing programs, they must pay attention to "program fidelity" (quality control). They should evaluate currently-funded programs to determine if benefits occur and if they exceed costs. They should develop the specialized knowledge needed to keep abreast of the latest research from around the world.

It is time for the world to face up to the fact that preventing as many children and youth as possible from starting to smoke cigarettes is feasible and worthwhile, both economically and in terms of improved health of the population.

## Competing interests

The author is married to the developer and owner of the Positive Action program, one of the programs discussed in this paper.

## Supplementary Material

Additional File 1 **Table S1.** Short and Long-term Effects of Seven Selected Social Influence Programs with Follow-up Into High School. Click here for file

Additional File 2 **Table S2.** Other Programs of Interest. Click here for file
