# Supplementary material for: School-based smoking prevention programs with the promise of long-term effects
Source: Tob Induc Dis. 2009 Mar 26;5(1):6. doi: 10.1186/1617-9625-5-6 (PMC2667427; doi:10.1186/1617-9625-5-6)
Supplement: Additional File 1 — Table S1. Short and Long-term Effects of Seven Selected Social Influence Programs with Follow-up Into High School. [file 1617-9625-5-6-S1.doc]

| **Table S1: Short and Long-term Effects of Seven Selected Social Influence Programs with Follow-up Into High School^a^** | | | | | | | | | | | | | | | |
| --- | --- | --- | --- | --- | --- | --- | --- | --- | --- | --- | --- | --- | --- | --- | --- |
|  |  |  |  |  |  |  |  |  |  |  |  |  |  |  |  |
| Investigator | Project name | Design^b^ | N classes | Length | Type^c^ | Grades | Grade at last follow-up | Short-term effect size (%)^d^ | | |  | Long-term effect size (%)^d^ | | |  |
|  |  |  |  |  |  |  |  |  |  |  |  |  |  |  |  |
|  |  |  |  |  |  |  |  | Life | Month | Week | Av ES | Life | Month | Week | Av ES |
| **School only programs** | |  |  |  |  |  |  |  |  |  |  |  |  |  |  |
| Hansen^e^ | TAPP (Cohort 1) | NR-S | 15 | 1 yr | S | 7 | 10 |  | 26.2% |  | 26.2% | 18.3% | 19.1% |  | 18.7% |
| Botvin^f^ | Life Skills Training | R-S | 30 | 3 yrs | S | 7-9 | 12 |  |  | 8.9% | 8.9% |  | 19.7% | 20.4% | 20.0% |
| Elder^g^ | Project SHOUT | R-S | 18+ | 3 yrs | S+ | 7-9+ | 11 |  | 30.3% |  | 30.3% |  | 44.1% |  | 44.1% |
| **MEANS for school programs** | | | | | | | |  | 28.2% | 8.9% | **21.8%** | 18.3% | 27.6% | 20.4% | **27.6%** |
| **School plus community or mass media programs** | | | | | | | | | | | | | | | |
| Vartiainen^h^ | N Karelia | NR-C | 10+ | 2 yrs | S+C | 7-8 | 11 | 44.8% | 43.7% | 45.3% | 44.6% | 40.3% | 39.2% | 36.7% | 38.7% |
| Perry | Minnesota Class of 89 | NR-C | 17+ |  | S+C | 6-10 | 12 |  |  | 40.0% | 40.0% |  |  | 39.4% | 39.4% |
| Pentz ^i^ | MPP | PR-S | 15+ | 2 yrs | S+C | 6-7/7-8 | 9-10 |  | 40.9% | 34.1% | 37.5% |  | 18.0% |  | 18.0% |
| Flynn ^j^ | Vermont Mass Media | R-C | 22+ | 4 yrs | S+M | 5-8, 6-9 or 7-10 | 10-12 |  |  | 36.6% | 36.6% |  |  | 28.8% | 28.8% |
| **MEANS for School + Community or Media Programs** | | | | | | | | 44.8% | 42.3% | 39.0% | **39.7%** | 40.3% | 28.6% | 35.0% | **31.2%** |
| **OVERALL MEANS for all programs** | | | | | | | | 44.8% | 35.3% | 33.0% | **32.0%** | 29.3% | 28.0% | 31.3% | **29.7%** |
| a: All studies except Vartiainen (North Karelia, Finland) took place in the USA. | | | | | | | |  |  |  |  |  |  |  |  |
| b: R = Random, NR = nonrandom, PR = partial random, S = School, C = Community | | | | | | | |  |  |  |  |  |  |  |  |
| c: S = School only, S+ = School plus small media or family outreach, M = Mass Media, C = Community | | | | | | | | | | | | | | | |
| d: Percent relative improvement (RI) as either (%change in C - %change in P)/%C or (%C-%P)/%C, where P = Program condition and C = Control. Short-term effects are generally at the end of grade 8 or 9. | | | | | | | | | | | | | | | |
| e: Tobacco and Alcohol Prevention Project. The long-term effect for smoking in the past month was larger (42.9%) for students present at all waves of the study. | | | | | | | | | | | | | | | |
| f: Randomization was originally complete, but 6 program schools were dropped from analysis because of low implementation. The RI for high-implementation students at 12th grade was 37%. | | | | | | | | | | | | | | | |
| g: The effect reported is with half the high school students receiving a high-school intervention (2 newsletters and 1 phone call during grade 1), without it, effect size is only 17.1%, with it for all students, the effect size is 71.4%. | | | | | | | | | | | | | | | |
| h: At 3 years post HS the effect was 22.0% for the health educator (HE) condition and 37.3% for the teacher condition; at 10 years post HS the effect was 20% for the HE condition and 19.5% for the teacher condition. | | | | | | | | | | | | | | | |
| i: Midwestern Prevention Project. | | | | | | | | | | | | | | | |
| j: This study tests the difference between school plus mass media and school-only (there was no control group). | | | | | | | | | | | | | | | |
