# Supplementary material for: School-based smoking prevention programs with the promise of long-term effects
Source: Tob Induc Dis. 2009 Mar 26;5(1):6. doi: 10.1186/1617-9625-5-6 (PMC2667427; doi:10.1186/1617-9625-5-6)
Supplement: Additional File 2 — Table S2. Other Programs of Interest. [file 1617-9625-5-6-S2.doc]

| **Table S2: Other Programs of Interest^a^** | | | | | | | | | | | | | | | | | | | | | | | | | | | |
| --- | --- | --- | --- | --- | --- | --- | --- | --- | --- | --- | --- | --- | --- | --- | --- | --- | --- | --- | --- | --- | --- | --- | --- | --- | --- | --- | --- |
| Investigator | Project name | Design^b^ | | N classes | | Length | | Type^c^ | | | Grades | | Grade at last follow-up | | Short-term effect size (%)^d^ | | | | | | | | Medium-term effect size (%)^d^ | | | | |
|  |  |  |  |  |  |  |  |  |  |  |  |  |  |  | Life | | Month | | Week | | Av ES | | Life | | Week | | Av ES |
| Graham & Hansen^e^ | AAPT | NR-S | | 9 | |  | | S | | | 7 | | 11 | | 21.4% | | 26.2% | |  | | 23.8% | | 13.9% | |  | | 13.9% |
| Sussman^f^ | TNT | R-S | | 12 | | 2 yrs | | S | | | 7-8 | | 9 | | 34.4% | |  | | 64.3% | | 49.3% | | 30.4% | | 55.6% | | 43.0% |
| Walter^g^ | KYB | R-S | | 384 | | 6 yrs | | S+ | | | 4-9 | | 9 | |  | |  | |  | | 11.5% | | 36.6% | |  | | 36.6% |
| Bush^h^ | KYB | R-S | | 250 | | 4 yrs | | S+ | | | 4-7, 5-8 or 6-9 | | 7-9 | |  | |  | |  | | 49.5% | |  | |  | | 80.8% |
| Kellam^i^ | GBG | R-K | | 120 | | 2 yrs | | S | | | 1 | | 8 | |  | |  | |  | | 24.4% | | 20.07% | |  | | 20.07% |
| Storr/ Fur-Holden^j^ | GBG +ML | R-K | |  | | 1 yr | | S | | | 1 | | 8 | | 22.2% | |  | |  | | 22.2% | | 26.3% | |  | | 26.3% |
| Flay^k^ | PA - Hawai`i | R-S | | 560 | | 4 yrs | | S+ | | | 2-5 or 3-6 | | 5 or 6 | |  | |  | |  | | 30.0% | |  | |  | | 32.0% |
| Flay^k^ | PA - Chicago | R-S | | 420 | | 3 yrs | | S+ | | | 3-5 | | 5 | |  | |  | |  | | 29.0% | |  | |  | |  |
| Ausems^l^ |  | PR-S | | 3- | | 3mo | | S+ | | | 7 | | 9 | | 30.0% | |  | |  | | 30.0% | | 43.2% | |  | | 43.2% |
| Biglan^m^ | Project 16 | R-C | | 5+ | | 2 yrs | | S+C | | | 7-9 | | 7-9 | | 21.1% | |  | |  | | 21.1% | | 27.5% | |  | | 27.5% |
| **MEAN EFFECT SIZES** | | | | | | | | | | | | | | | 25.8% | | 26.2% | | 64.3% | | **29.1%** | | 28.3% | | 55.6% | | **35.9%** |
| a: All studies except Ausems (Netherlands) took place in the USA. | | | | | | | | | |  | |  | | |  | |  | |  | |  | |  | |  | |  |
| b: R = Random, NR = nonrandom, PR = partial random, K = Student, S = School, C = Community | | | | | | | | | | | | | | | | |  | |  | |  | |  | |  | |  |
| c: S = School only, S+ = School plus small media or family outreach, M = Mass Media, C = Community | | | | | | | | | | | | | | | | | | | | | | | | | | | |
| d: Percent relative improvement (RI) as either (%change in C - %change in P)/% change in C or (%C-%P)/%C, where P = Program condition and C = Control. Short-term effects are generally at the end of grade 8 or 9. | | | | | | | | | | | | | | | | | | | | | | | | | | | |
| e: Adolescent Alcohol Prevention Trial | | | | |  | |  | |  |  | |  | |  | |  | |  | |  | |  | |  | |  | |
| f: Towards No Tobacco | | |  | |  | |  | |  |  | |  | |  | |  | |  | |  | |  | |  | |  | |
| g: Know Your Body. Included parent communications. Effects are for saliva thiocyanate, a biological indicator of tobacco use. Long-term effects were reported only for one site (so value shown is half value reported at that site) and significant only for males (0% vs. 12%). | | | | | | | | | | | | | | | | | | | | | | | | | | | |
| h: A replication of KYB in Washington DC area schools. Results are saliva thiocyanate, short-term after 1 yr of intervention, long-term after 3 years. | | | | | | | | | | | | | | | | | | | | | | | | | | | |
| i: Good Behavior Game. Initially 3 10-minute classes per week in grade 1, increasing in duration and frequency during grades 1 and 2. Short-term effects are for "Problem behavior" at the end of grade 2. Smoking effects were significant only for boys. | | | | | | | | | | | | | | | | | | | | | | | | | | | |
| k: Good Behavior Game plus Mastery Learning in grade 1 only. Very similar results were obtained in a condition that added family partnerships. Short term results are at the end of grade 7. | | | | | | | | | | | | | | | | | | | | | | | | | | | |
| k: Consists of about 140 15-minute classes per elementary grade. The medium-term effect entered in the table is the effect in middle school for students exposed to PA in elementary school estimated from a different (quasi-experimental) study (see text). | | | | | | | | | | | | | | | | | | | | | | | | | | | |
| l: Three computer-tailored letters based on the stages of change model (TTM). A classroom plus letters condition produced similar or worse results. Reported results are for baseline never smokers. Results were similar for baseline smokers. | | | | | | | | | | | | | | | | | | | | | | | | | | | |
| m: Multiple cross-sectional design, where successive cohorts of 7th and 9th grade students were surveyed. | | | | | | | | | | | | | | | | | |  | |  | |  | |  | |  | |
